# Clinical tolerability of artesunate-amodiaquine versus comparator treatments for uncomplicated falciparum malaria: an individual-patient analysis of eight randomized controlled trials in sub-Saharan Africa

**DOI:** 10.1186/1475-2875-11-260

**Published:** 2012-08-02

**Authors:** Julien Zwang, Grant Dorsey, Abdoulaye Djimdé, Corine Karema, Andreas Mårtensson, Jean-Louis Ndiaye, Sodiomon B Sirima, Piero Olliaro

**Affiliations:** 1 Drugs for Neglected Diseases Initiative (DNDi), Geneva, Switzerland; 2 Department of Medicine, University of California, San Francisco, CA, USA; 3 Malaria Research and Training Center, Department of Epidemiology of Parasitic Diseases, Faculty of Medicine and Pharmacy, University of Science, Techniques and Technology of Bamako, Bamako, Mali; 4 Malaria & Other Parasitic Diseases Division-RBC, Ministry of Health, Rwanda, Africa; 5 Infectious Diseases Unit, Department of Medicine, Karolinska University Hospital, Karolinska Institutet, Stockholm, Sweden; 6 Division of Global Health (IHCAR), Department of Public Health Sciences, Karolinska Institutet, Stockholm, Sweden; 7 Department of Parasitology, Faculty of Medicine, Cheikh Anta Diop University, Dakar, Senegal; 8 Centre National de Recherche et de Formation sur le Paludisme, Ministère de la Santé, Ouagadougou, Burkina Faso; 9 UNICEF/UNDP/WB/WHO Special Programme for Research & Training in Tropical Diseases (TDR), Geneva, Switzerland; 10 Centre for Tropical Medicine and Vaccinology, Nuffield Department of Medicine, University of Oxford, Churchill Hospital, Oxford OX3 7LJ, UK

**Keywords:** Malaria, *Plasmodium falciparum*, Safety, Tolerability, Adverse event, Treatment-emergent adverse event, Artesunate-amodiaquine, Treatment, Randomized controlled trial, Sub-Saharan Africa

## Abstract

**Background:**

The widespread use of artesunate-amodiaquine (ASAQ) for treating uncomplicated malaria makes it important to gather and analyse information on its tolerability.

**Methods:**

An individual-patient tolerability analysis was conducted using data from eight randomized controlled clinical trials conducted at 17 sites in nine sub-Saharan countries comparing ASAQ to other anti-malarial treatments. All patients who received at least one dose of the study drug were included in the analysis. Differences in adverse event (AE) and treatment emergent adverse event (TEAE) were analysed by Day 28.

**Results:**

Of the 6,179 patients enrolled (74% <5 years of age), 50% (n = 3,113) received ASAQ, 20% (n = 1,217) another ACT, and 30% (n = 1,849) a non-ACT (combination or single-agent) treatment. Overall, 8,542 AEs were recorded. The proportion of patients experiencing at least one gastro-intestinal AE on ASAQ was 43% (and higher than that with artemether-lumefantrine and dihydroartemisinin-piperaquine at two sites), and was 23% for any other AEs (not different from other treatments). Specifically, the risk of diarrhoea, vomiting, cough and weakness was lower with artemether-lumefantrine; artemether-lumefantrine and dihydroartemisinin-piperaquine carried a higher risk of pruritus, chloroquine-SP had a higher risk of nausea. Parasitological recurrence increased the risk of occurrence of any AE. No other difference was detected. Comparing AE to TEAE in patients who had pre-treatment occurrence and grades of intensity recorded, AEs were significantly more related to the pre-treatment prevalence of the symptom (p = 0.001, Fisher test); AEs overestimated TEAEs by a factor ranging from none to five-fold. The overall incidence of serious AEs (SAEs) with ASAQ was nine per 1,000 (29/3,113) and mortality was one per 1,000 (three deaths, none drug-related); both were similar to other treatments.

**Conclusion:**

ASAQ was comparatively well-tolerated. Safety information is important, and must be collected and analysed in a standardized way. TEAEs are a more objective measure of treatment-induced toxicity.

## Background

Artemisinin-containing combination therapy (ACT) is the World Health Organization (WHO) recommended first-line treatment of acute uncomplicated *Plasmodium falciparum* malaria [1]. One such treatment is artesunate plus amodiaquine (ASAQ), which has been commercialized and dispensed as either loose or co-blistered individually formulated products (loose ASAQ), and, more recently as a fixed-dose coformulation (ASAQ FDC: first market authorization in 2007, WHO prequalified in 2008, included in the Essential Medical List (EML) in 2011).

Worldwide, ASAQ (in its various formulations) is the second most widely used ACT. The volumes of ASAQ FDC have increased from less than one million treatment courses in 2007 to 41 million in 2010 [1], of which some six million treatments in 2008, 25 million in 2009 and over 45 million in 2010 were purchased via international organizations [2,3]. Over 23 million treatments of ASAQ Whintrop^TM^ FDC had been ordered by 2012 for the private sector of the seven African countries (Ghana, Kenya, Nigeria, Niger, Tanzania, Madagascar, Uganda) included in the AMFm (Affordable Medicines Facilities - malaria) pilot phase (from 2010 to February 2012) [4]. By 2011 around 120 million of ASAQ FDC treatments had been distributed in 21 countries [5].

Although ACT is regarded as highly effective and generally well tolerated, safety remains largely under-reported. Synthesizing available information using individual patient data for randomized controlled trials (RCTs) can help better define treatments in terms of risk-profiling and risk-management. There is also a real need to develop a standardized system for generating and analysing tolerability data from anti-malarial drug efficacy studies; while an excellent standardized system for efficacy analyses exists, there is no equivalent for safety and tolerability.

For this study, relevant information was retrieved from randomized controlled trials (RCTs) identified through a systematic review for which individual patient data were made available. This analysis included a majority of children under five years old from sub-Saharan Africa since they represent 86% of the death toll due to malaria in the world [1].

## Methods

### Study endpoints

The analysis of tolerability was by intent-to-treat (ITT), including all participants who were randomized to the study medications and took at least one dose and followed-up to Day 28. The primary study endpoint in all the RCTs included in the analysis was efficacy. Follow-up ceased at the time of parasitological failure (either primary or recurrence), protocol violation, loss to follow-up, and no tolerability data were recorded thereafter. Signs or symptoms were recorded at enrolment (Day 0) and tolerability outcomes were recorded post-treatment from Day 1 to Day 28 according to study procedures.

### Study sites, design and patients

The studies were identified through a systematic review of clinical trials and personal contacts, regardless of language or publication status (published, unpublished, in press, reports). Published studies were identified through electronic searches up to April 2007 of MEDLINE, EMBASE, LILACS, the Cochrane Infectious Diseases Group's trials register and the Cochrane Central Register of Controlled Trials (CENTRAL) using the search terms: malaria, amodiaquine, artesunate, and artemisinin. To be included, studies were to be RCTs conducted in Africa comparing any formulation of ASAQ to any other single or combination treatment of uncomplicated falciparum malaria with follow-up of at least 28 days [6].

For the studies meeting these criteria, investigators were contacted to provide individual patient data and datasets received were examined for inclusion.

### Design of the included studies

Trials were all randomized comparative: one was double-blinded [7], four were open label [8-11], and three were single blinded [12-14]. All studies used methods to conceal allocation, gave treatment under supervision and followed patients up for 28 days. More details on the studies methodological quality can be found elsewhere [15].

### Tolerability outcomes

The tolerability outcome measure was any treatment adverse event (AE) and treatment-emergent adverse event (TEAE) occurring within 28 days of starting treatment. In each of the studies included, signs or symptoms were actively screened during follow-up [7-14].

An AE was defined as any sign or symptom occurring after the start of treatment (first drug intake), irrespective of whether that sign or symptom was present at baseline or not, of its severity and drug-event relationship.

A TEAE was defined as the worsening of the condition - ie any occurrence of an abnormal condition as compared to baseline in patients who either had a normal condition pre-treatment or an abnormal condition that was of lower intensity than that recorded post-treatment (worst grade reported at any time, irrespective of whether it improved later). Intensity was graded nought to four (absent, mild, moderate, severe, very severe) - this analysis was only possible in the sub-group of patients from the sites who graded signs and symptoms; in two other studies [7,9] grades were recorded during the follow-up but not at enrolment, therefore TEAEs could not be analysed.

Serious adverse events (SAEs) were defined as events that were fatal, life threatening or required admission to hospital, irrespective of drug-effect relationship. Death was also reported separately. The incidence of AEs and TEAE were calculated for each study and treatment arm after standardizing AE terminology (i) translating French into English language (ii) including redness, swelling, burning, itching, rash skin symptom (iii) grouping weakness with fatigue and asthenia.

The disposition of the two components of the ASAQ combination is very different; AS is rapidly absorbed and eliminated (half-life <1 hour), while the estimated median (range) elimination half-life of desethyl-AQ (AQ active metabolite) is around nine (seven to 12) days. The same is also true for the other ACT. Therefore most of the AEs will likely happen before Day 28.

### Statistical analysis

Data were standardized for an ITT analysis. Incidences (proportions of patients experiencing an event) were calculated for each treatment group and each sign or symptom and defined as the number of patients reporting the event divided by the number of patients initially at risk (exposed). As all studies did not provide information on the exact day of the event, it was not possible to calculate the overall time-to-event expressed as person-day to calculate incidence rate ratios, and AEs and TEAEs were analysed as a binary variable occurring per patient. The risks of experiencing AEs/TEAEs with ASAQ compared to other drugs were measured by using multivariate logistic regression. A random intercept for each site was included when the Lagrange multiplier (LM) test [16] was significant for heterogeneity in multivariate logistic regression. Since the day or the date of occurrence of the adverse event was not available for around 2,000 patients with information on event intensity, and in order to control for differences in efficacy and account for differences in the duration of follow-up between treatment groups which could be due to potential differences in treatment efficacy, the last day of observation was included in the logistic regression models with a random effect on the site. The adjusted risks (AOR) of these AEs and TEAEs were assessed according to the patients’ age (continuous in year); parasitaemia at enrolment (continuous, log-transformed); the last day of observation (continuous in day) and the treatment (categorical).

An additional analysis comparing AE and TEAE was included using the sub-group of patients with sign or symptom recorded and graded both pre- and post-treatment. Categorical data were compared using the chi-square or the Fisher exact test. The non-normally distributed correlation used the Spearman test and compared by the Fisher test. Confidence intervals were calculated at 95% (95%CI), and comparisons considered significant when P < 0.05. Data were analysed using Stata v10 (Stata Corp.).

### ASAQ treatment regimens

The majority of the patients were treated with individually formulated (loose) AS and AQ. The target dose was AS 12 mg/kg over three days and AQ 30 mg/kg over three days except in Uganda where AQ was given at 25 mg/kg (Day 0: 10 mg/kg, Day 1: 10 mg/kg, Day 2: 5 mg/kg). The loose combinations ASAQ were given based on body weight, while in two studies the fixed combination (FDC) ASAQ was given based on age and weight [11,14]. The loose combination was administered once-a-day, while the FDC was given either once- or twice-a-day [14].

### Comparator treatment regimens

ACTs: AL (20 mg artemether/120 mg lumefantrine given according to weight as one (5–14 kg), two (15–24 kg), three (25–34 kg), and four (≥ 35 kg) tablets given twice daily co-administrated with fat for 3 days); DP (2.3 mg/kg/day dihydroartemisinin and 18.4 mg/kg piperaquine for three days); AS + SP (AS 4 mg/kg/day; SP 25 mg/kg of sulphadoxine and 1.25 mg/kg/of pyrimethamine administered in a co-formulated tablet SP as a single dose);

Non-ACTs: AQ + SP (AQ 10 mg/kg/day for three days and SP 25 mg/kg of sulphadoxine and 1.25 mg/kg/of pyrimethamine administered in a co-formulated tablet SP as a single dose); CQ (25 mg/kg chloroquine over three days) and SP; AQ mono-therapy (10 mg/kg/day for three days); AS mono-therapy (AS 12 mg/kg over five days).

### Ethical issues

All studies have been approved by the relevant ethics and institution review committees [7-14].

## Results

### Characteristics of included studies

A total of 6,179 patients was enrolled in eight studies conducted at 17 sites in nine countries (Table 1):

**Table 1:** Number and age of patients included in the analysis

**Site and Country**	**Reference**	**Age (year)**			**ASAQ**	**AL**	**AQ**	**AQ + SP**	**AS + SP**	**AS**	**CQ + SP**	**DP**	**Total**
		**Median**	**Mininum**	**Maximum**									
Burkina Faso	[9]	2.0	0.5	5	750								750
Cameroon	[7]	6.2	1.0	65	110	56							166
Gabon	[8]*	5.5	1.3	11	108		110						218
Madagascar	[9]	7.4	1.4	53	119	60							179
Mali, Boulouga	[7]	4.7	0.9	24	135	68							203
Mali, Bancoumana	[10]	3.0	0.6	56	252				249	252			753
Rwanda, Kicukiro	[11]*	3.3	1.0	5	74			74				75	223
Rwanda, Mashesha	[11]*	3.0	1.1	5	89			93				87	269
Rwanda, Rukara	[11]*	2.1	1.0	5	89			91				90	270
Senegal, Keur-Socé	[7]	9.0	1.0	15	264	128							392
Senegal, Mlomp	[11]*	5.0	0.9	64	160		160						320
Uganda, Apac	[13]*	1.8	0.5	47	174			183			185		542
Uganda, Jinja	[13]*	3.6	0.5	65	189			186			168		543
Uganda, Tororo	[13,14]*	1.6	0.5	56	398	204		181			166		949
Zanzibar, Kivungue	[12]	2.5	0.5	7	148	149							297
Zanzibar, Micheweni	[12]	2.1	0.5	5	54	51							105
Total		3.0	0.5	65	3113	716	270	808	249	252	519	252	6179

Legend: * studies including grading of intensity AQ, amodiaquine; AL, artemether-lumefantrine; AS, artesunate; DP, dihydroartemisinin-piperaquine; CQ, chloroquine; SP, sulphadoxine-pyrimethamine.

(i) Two multi-country studies (n = 1,879): one comparing ASAQ FDC (n = 628, of whom 313 were administered once and 315 twice daily) to AL (n = 312) in Mendong-Cameroon; Tsiroanomandidy-Madagascar, Bancoumana-Mali, Mlomp-Senegal [7]; and the other comparing loose ASAQ (n = 268) to AQ (n = 270) in Lambaréné-Gabon and Oussouye-Senegal [8].

(ii) Two single centre study were conducted in Pouytenga-Burkina Faso comparing ASAQ fixed dose (FDC, n = 375) to the loose ASAQ combination (n = 375) [9] and in Boulouga-Mali to compare loose ASAQ (n = 252), AS (n = 252) and AS + SP (n = 249) [10].

(iii) Two studies conducted in a single country with multiple sites in Rwanda-Mashesha, Rukara, and Kicukiro comparing loose ASAQ (n = 252) to AQ + SP (n = 258) and DP (n = 252) [11]; and in Zanzibar-Kivunge, and Micheweni to compare ASAQ (n = 202) to AL (n = 200) [12].

(iv) Two different studies in Uganda used the same protocol in three common sites: Apac, Jinja and Tororo comparing loose ASAQ (n = 761) to AL (n = 204), AQ + SP (n = 550), CQ + SP (n = 519) [13,14].

Individually, the sites enrolled between 174 and 949 patients treated with either ASAQ, which constituted 50% (n = 3,113) of the total patient population, or a comparator: another ACT (20%, 1,217/6,179) or non-ACT (30%, 1,849/6,179) (Table 1). Studies took place from February 1999 in Gabon [8] to December 2006 in Senegal [7]; 74% of the patients (4,579/6,179) were children aged between four and 59 months.

In all cases, allocation was concealed until after a patient had given written consent to participate in the study. All treatments were supervised over the three-day course. According to WHO recommendations, after each dose, children were observed for 30 min and the dose was re-administered if vomiting occurred. Children who repeatedly vomited their first dose of study medication were excluded from the study and referred for further management.

### Adverse events (AEs)

**Figure 1:**
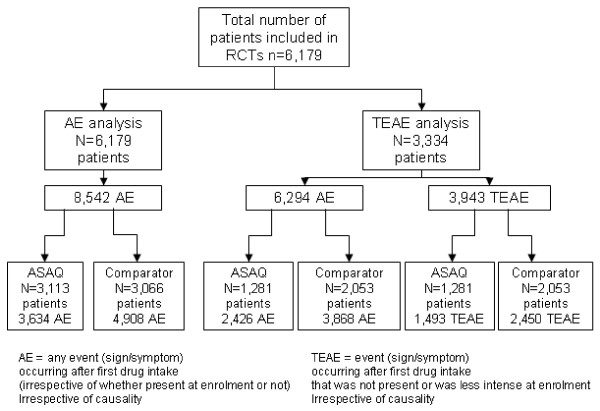
Flow chart of patients included in the analysis.

Overall 8,542 AEs of 14 different types were recorded occurring in 6,179 patients receiving at least the first treatment dose (Figure 1) – each individual not having or having one or multiple AEs; specifically, 3,634 AEs occurred in 3,113 patients treated with ASAQ and 4,908 AEs occurred in 3,066 patients treated with a comparator. The overall median proportion of patients on ASAQ experiencing at least one gastro-intestinal (GI) AE was 43% (ranging from 3% to 91%), or at least one other AE was 23% (ranging from 1% to 80%).

Using random effects multivariate logistic regression, the risk of experiencing any GI AE was lower with AL (AOR 0.67, 95%CI 0.51-0.87, p = 0.003) and DP (AOR 0.66, 95%CI 0.45-0.97, p = 0.035) than with ASAQ, resulting from differences seen in two sites only; the occurrence of GI AEs was significantly related to the risk of parasitological recurrence (AOR 1.21, 95%CI 1.03-1.41, p = 0.019); older patients were at lower risks for GI (AOR 0.98, 95%CI 0.97-0.99, p = 0.032).

 For other AEs, no difference between treatment groups was detected. The risk was significantly related to the risk of parasitological recurrence (AOR 1.48, 95%CI 1.24-1.76, p = 0.001), age (older patients were at higher risks for other AEs (AOR 1.03, 95%CI 1.01-1.04, p = 0.001), and duration of follow-up (AOR 1.01, 95%CI 1.01-1.02, p = 0.037).

There was no difference between the loose and fixed dose ASAQ combination; no other risk was detected.

### Gastro-intestinal (GI) AEs

**Figure 2:**
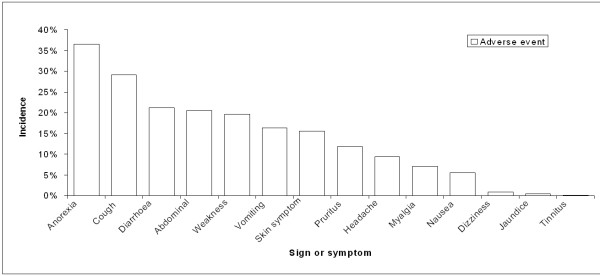
Adverse events (AE) incidence, ASAQ groups.

In patients treated with ASAQ (Table 2, Figure 2), the incidence of anorexia was 36% (789/2,164), diarrhoea 21% (526/2,483), abdominal pain 20% (280/1,368), vomiting 16% (479/2,937), and nausea 6% (116/2,104).

**Table 2:** Adverse event (AE) incidence in ASAQ groups

**Sign or Symptom**	**Adverse Event (AE)**		
	**Number of patients with AE**	Total number of patients *	**Incidence**
Anorexia	789	2164	36%
Cough	509	1747	29%
Diarrhoea	526	2483	21%
Abdominal	280	1368	20%
Weakness	539	2749	20%
Vomiting	479	2937	16%
Skin symptom	118	759	16%
Pruritus	193	1636	12%
Headache	164	1739	9%
Myalgia	23	324	7%
Nausea	116	2104	6%
Dizziness	19	2150	1%
Jaundice	4	759	1%
Tinnitus	1	828	0%

* The total number of patients was different since different signs or symptoms were screened across the studies.

In Rwanda [11], 11.5% of patients vomited ASAQ, which was not different from 9.9% for DP (p = 0.565) but lower than with AQ + SP (19.5%, p = 0.002). In Burkina Faso [9], 1.9% patients (14/750) were withdrawn from the study for drug-induced vomiting, eight (2.1%) and six (1.6%) with FDC and loose ASAQ, respectively (*p* = 0.59). In Mali [10], there was no difference in the incidence of vomiting between ASAQ and AS/SP or AS. In a multi-centre study [8], six patients in the loose ASAQ group and five in the AQ group developed early, drug-induced vomiting, necessitating alternative treatment. In the other multi-centre study [7], 13% (122/941) of the patients vomited within the first half-hour after treatment administration from Day 0 to Day 2 (no difference between ASAQ and AL). In Uganda and Zanzibar drug vomiting and other later vomiting were reported together [12-14].

**Figure 3:**
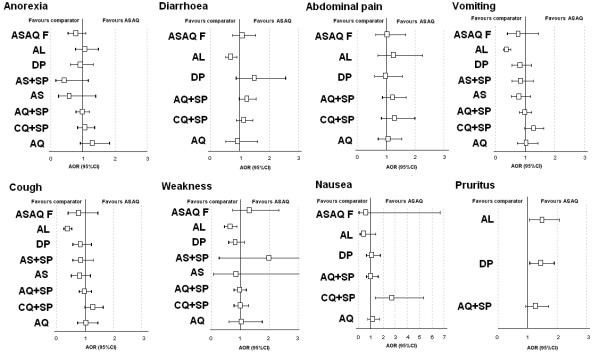
**Adverse events (AE) forest plots, ASAQ vs. comparator treatments. **AOR, adjusted odds ratio for age, log parasitaemia, parasitological reappearance, and treatments, randomized by site; CI, confidence interval; AQ, amodiaquine; SP, sulfadoxine-pyrimethamine; DP, dihydroartemisinin-piperaquine; CQ, chloroquine; AS, artesunate; AL, artemether-lumefantrine; ASAQ F, artesunate amodiaquine fixed dose combination.

Overall (all treatment groups), multivariate analysis for each GI AE showed that compared to ASAQ (Figure 3), patients treated with AL were at lower risk of diarrhoea (AOR 0.68, 95%CI 0.52-0.90, p = 0.006, resulting from a significant difference in Zanzibar) and vomiting (AOR 0.36, 95%CI 0.25-0.52, p = 0.001, resulting from significant differences in patients treated in Uganda and Zanzibar), while patients treated with CQ + SP were at higher risks of nausea (AOR 2.67, 95%CI 1.35-5.27, p = 0.005).

Other risk differences were related to age: older patients were at lower risk for vomiting, anorexia and diarrhoea, and at higher risk for abdominal pain (p < 0.005 for all comparisons); the risk of anorexia (AOR 1.28, 95%CI 1.08-1.52, p = 0.005) was higher in patients with a parasitological recurrence as well as the risk of diarrhoea (AOR 1.28, 95%CI 1.07-1.54, p = 0.007), which increased over time (AOR 1.02, 95%CI 1.01-1.04, p = 0.001).

### Other AEs

The incidence of headache on ASAQ was 9% (164/1,739) increasing with age (AOR 1.21, 95%CI 1.14-1.28, p = 0.001) and higher in patients treated with CQ + SP (AOR 1.87, 95%CI 1.13-3.09, p = 0.015) than ASAQ. Dizziness was infrequent (1%) in all groups. One case (in 848) of mild tinnitus was reported with loose ASAQ on the day of recrudescence (Day 21). Patients treated with CQ + SP (4%, 7/160) were at higher risks for this hearing disturbance compared to ASAQ (AOR 37.81, 95%CI 3.80-376.69, p = 0.001). Romberg test and nystagmus were negative in all the patients screened (398 and 1592, respectively). The incidence of pruritus was 19% (193/1636) with ASAQ; the risk was comparatively higher with AQ + SP (AOR 1.43, 95%CI 1.08-1.90, p = 0.013) and AL (AOR 1.48, 95%CI 1.06-2.06, p = 0.021). The incidence of skin conditions (redness, swelling, burning, itching, rash) was 16% (118/759) on ASAQ (not different from other groups).

The incidence of weakness with ASAQ was 20% (539/2,749), and the risk was higher in patients with a parasitological failure (AOR 1.78, 95%CI 1.46-2.17, p = 0.001) and lower in patients treated with AL, resulting from a significant incidence difference found in Madagascar (AOR 0.62, 95%CI 0.44-0.88, p = 0.006) (Figure 3). The incidence of myalgia (muscle pain) was 8% (17/217) with no difference between treatment groups.

Cough was the second most frequent AE reported for ASAQ (29%, 509/1,747). No difference between treatments was detected, but older patients were at lower risks (AOR 0.92, 95%CI 0.90-0.95, p = 0.001), and the risk increased during the follow-up (AOR 1.05, 95%CI 1.03-1.07, p = 0.001). Jaundice was infrequent (<1%) in all groups.

### AE and TEAE

In this sub-group, 3,943 TEAEs or 6,294 AEs occurred in 3,334 patients who had received at least one treatment dose (Figure 1). Some 1,493 TEAEs or 2,426 AEs occurred in 1,281 patients treated with ASAQ and 2,450 TEAEs or 3,868 AEs in 2,053 patients treated with comparator drugs.

**Figure 4:**
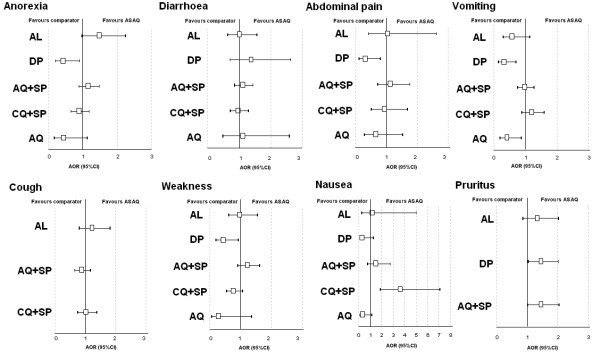
**Treatment emergent adverse event (TEAE) forest plots, ASAQ vs. comparator treatments. **AOR, adjusted odds ratio for age, log parasitaemia, parasitological reappearance, time, and treatments, randomized by site; CI, confidence interval; AQ, amodiaquine; SP, sulfadoxine-pyrimethamine; DP, dihydroartemisinin-piperaquine; CQ, chloroquine; AS, artesunate; AL, artemether-lumefantrine.

Using multivariate analysis, compared to ASAQ, patients treated with DP were at lower risk for GI AEs or TEAEs (AOR 0.66, 95%CI 0.45-0.97, p = 0.035, AOR 0.50, 95%CI 0.32-0.79, p = 0.003, respectively), while patients treated with AQ were at lower risk for GI TEAEs (AOR 0.57, 95%CI 0.33-0.98, p = 0.041) but not AEs (p = 0.927). For any other AEs or TEAEs, no difference between treatments was observed. Older patients were at significant lower risks for gastro-intestinal AE and TEAE. Between-treatment comparisons for each TEAE are presented using forest plots in Figure 4.

### Difference between AEs and TEAEs on ASAQ treatment

**Figure 5:**
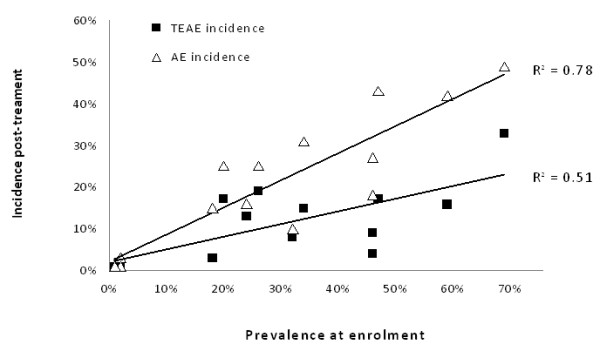
**Relationship between the condition before treatment and after treatment using AE or TEAE definition, ASAQ groups. **AE, adverse event; TEAE, treatment emergent adverse event.

The incidence of AEs and TEAEs following ASAQ treatment is presented in Table 3, and Figure 5. Both AEs and TEAEs incidences were correlated with the prevalence of the respective signs/symptoms at enrolment (r = 0.86, p = 0.001, r = 0.66, p = 0.001, respectively, Spearman test), which was comparatively stronger for AEs than TEAEs (p = 0.001, Fisher test). There was also a significant relationship between admission at enrolment and the difference in incidence between AE and TEAE (r = 0.85, p = 0.001, Spearman test).

**Table 3:** Incidence of treatment emergent adverse events (TEAEs) and adverse events (AEs) patients treated with ASAQ

**Sign or symptom**		**Total number of patients**	**Prevalence at enrolment**	**TEAE**		**AE**		**AE vs TEAE**
				**n**	**Incidence**	**n**	**Incidence**	**Relative difference**
Gastro-intestinal	Anorexia	1279	47%	217	17%	551	43%	154%
	Abdominal	740	46%	66	9%	202	27%	206%
	Vomiting	1277	34%	188	15%	392	31%	108%
	Diarrhoea	1278	20%	214	17%	315	25%	47%
	Nausea	726	18%	22	3%	107	15%	386%
Neurological	Headache	736	46%	27	4%	132	18%	388%
	Dizziness	519	2%	3	1%	15	3%	399%
	Tinnitus	200	1%	1	1%	1	1%	0%
Dermatological	Pruritus	756	26%	141	19%	192	25%	36%
	Skin symptom	759	24%	97	13%	118	16%	22%
Other	Cough	759	69%	248	33%	371	49%	50%
	Weakness	1119	59%	176	16%	470	42%	167%
	Myalgia	216	32%	17	8%	21	10%	24%
	Jaundice	759	2%	4	1%	4	1%	0%

* The total number of patients was different since different signs or symptoms were screened across the studies.

The comparison between prevalence of signs/symptoms on admission and AE and TEAE intended to quantify the difference in appreciation of drug tolerability when reporting TEAEs or AEs (i.e. whether accounting for pre-treatment intensity of signs/symptoms).

Table 3 presents the prevalence of signs/symptoms at enrolment and compares their incidence post-treatment when reported as AEs or TEAEs. Except tinnitus and jaundice, all other events were more frequent by a factor of 22%-399% when reported as AEs than TEAEs.

For GI events, the prevalence of diarrhoea at enrolment was 20%; the incidence of AEs and TEAEs was 25% and 17% respectively, and the difference between the two was +47% (meaning that 8%, n = 101, of the patients had at least one episode of diarrhoea with the same or decreasing intensity at any time during follow-up compared to pre-treatment). For nausea (18% at enrolment), the difference between AE and TEAE was +386%.

For neurological effects, headache was common at enrolment (46%) and decreased post-treatment (incidence of AE = 18%, TEAE = 3%, difference +388%). Both dizziness and tinnitus were infrequent at enrolment and after treatment. For dermatological symptoms, the prevalence of pruritus was 26% pre-treatment, and the incidence of AEs and TEAEs was 25% and 19% (difference +36%), meaning that 6% of the patients had the same or decreasing symptom intensity during follow-up.

Regarding other signs or symptoms, cough was the most frequent at enrolment (69%); 16% (33%, TEAE; 49%, AE) of patients had subsequently decreasing or stable intensity. Weakness was also very frequent at enrolment (59%) with 26% of patients with a decreasing or stable intensity post-treatment. Muscle pain was quite frequent at enrolment (32%), and there was no significant difference between TEAE (8%) and AE incidence (10%); most of the patients developing muscle pain after treatment did not have that symptom at enrolment, while others recovered. Jaundice was infrequent (<1%, 4/759).

### Deaths

Overall six deaths occurred in the studies, corresponding to a mortality of one per 1,000 (3/3,113) with ASAQ, as well as with comparators (3/3,066). None of them was attributed to the study treatment. In Senegal [7] two patients died: one who received ASAQ had lung infection and anaemia on Day 3; the other one received AL group and died of fatal coma of unknown cause on Day 1.

Two male patients in Pouytenga-Burkina Faso, recruited for uncomplicated malaria and treated with the loose and FDC ASAQ respectively, died of severe cerebral malaria occurring after the first day of treatment [9]. One patient (nine months old, 6 kg, 38°C, 1,651 pct/μL, Hb: 12.3 g/dL, and no gametocyte at enrolment) died of severe malaria at home. According to the parents, apart from fever, no other symptom was observed and no other drug was given. The other patient (25 months old, 11 kg, 11,224 pct/μL, Hb: 6.8 g/dL) was hospitalized at night time with hyperpyrexia (39°C). He had upper limbs hypertonia, was unconscious and convulsed, and died in the afternoon after receiving parenteral quinine, diazepam, phenobarbitol, and noramidopyrine. The death was diagnosed as cerebral malaria.

In Uganda [13], two patients died: one of suspected severe malnutrition and the other one of congestive heart failure caused by a suspected congenital heart defect (both patients had received AQ + SP).

No deaths occurred in the studies included in the studies conducted in Gabon and Senegal [8] Mali [10], Rwanda [11], Zanzibar [12], or in Uganda, Tororo [14].

### Other serious adverse events (SAEs)

The incidence of other SAEs (except deaths) was nine per 1,000 (29/3,113) with ASAQ and six per 1,000 (18/3,066) with comparators (p = 0.124).

In Burkina-Faso [9], SAEs occurred in eight patients within the first three days of treatment except for one patient, who had gastroenteritis requiring hospitalization for rehydration on Day 11. With ASAQ FDC these were: two patients with convulsions (severe malaria) and one child with gastroenteritis. With the loose ASAQ, the SAEs were: one patient with convulsions and anaemia (severe malaria), one patient with severe prostration (severe malaria), and two patients with acute respiratory distress (consistent with either malaria-induced metabolic acidosis or pneumonia). All SAEs were considered unrelated to the study drugs.

In Rwanda [11] one seizure with ASAQ, and one with AQ + SP were reported.

In Uganda [13] 20 SAEs were reported in 16 patients (four loose ASAQ, four CQ + SP, eight AQ + SP, p = 0.40). SAEs included anaemia (two AQ + SP, one loose ASAQ), convulsion (one CQ + SP, two AQ + SP, one loose ASAQ), dehydration (one loose ASAQ), oedema (one AQ + SP), malnutrition (one CQ + SP), mental status change (two AQ + SP), respiratory distress or infection (one CQ + SP, two AQ + SP, one loose ASAQ), vomiting (one CQ + SP), and weakness (one AQ + SP).

In Uganda [14] SAEs occurred in two participants: one child treated with ASAQ developed pneumonia on Day 27, requiring hospitalization (unrelated to study drug), while a second participant, treated with AL, experienced a convulsion on Day 0 judged to be unlikely to be related to the study medication).

In Mali [10], two SAEs were reported: one case of severe anaemia (AS) and one case of respiratory distress and severe anaemia (ASAQ). Both patients were referred to the Regional Hospital and recovered fully.

In a multi-centre study [7], four patients reported SAEs after taking ASAQ. One case of severe anaemia required hospitalization (related to treatment according to the investigator). This event occurred on Day 7 of follow-up and the patient recovered after hospitalization. Three patients in the ASAQ group discontinued the study due to SAEs (persistence of severe vomiting, fatigue, vertigo and asthenia). These events were considered by the investigator to be probably related to treatment. All patients recovered.

In Senegal and Gabon [8], three patients reported SAEs after AQ or loose ASAQ: two patients had convulsions; one had asthma, one vomiting after the first drug intake, and one had gastroenteritis. All SAEs were regarded as unrelated to study drugs.

In Zanzibar [12], nine patients (seven treated with ASAQ group and two with AL) developed clinically suspected severe malaria and received rescue treatment. These SAEs were associated with worsening conditions and were thus not attributed to the study drugs.

## Discussion

While not exhaustive, this is so far the largest dataset of individual-patient tolerability data compiled on an ACT (ASAQ); it includes a sizeable number of patients (nearly 6,200, enrolled in randomized controlled trials, almost equally split between ASAQ and comparators groups) with tolerability outcomes (adverse events, AEs), and is representative of the spectrum composition of malaria patients (it comprises mostly children under five years of age (74%) from areas of moderate to high intensity of malaria transmission of nine sub-Saharan African countries). In more than half of these patients (over 3,300), events were recorded pre-treatment and graded in terms of intensity, which allowed identifying and analysing treatment emergent adverse events (TEAEs).

Both efficacy and tolerability information is essential to guide treatment policy. However, while millions of ACT treatments are given every year, and thousands of patients are enrolled in trials with ACT, little tolerability information is available. Tolerability data are often cursorily presented in papers and difficult to standardize and summarize. The availability of individual patient data and the use of standardized methods for analysis (including [17]) made it possible both to draw generalizable conclusions and to identify site-specific differences.

Over 3,100 of these patients received ASAQ formulated as different products (of whom over 2,100 were treated with the loose or co-blistered and 1,000 with the fixed dose combination) by various manufacturers. Therefore, the conclusions reported here do not relate to an individual drug product, but rather the collective tolerability profile of ASAQ. Overall, the risk of experiencing an AE was not different in patients exposed to ASAQ or other forms of ACT and non-ACT, with some exceptions.

This analysis also provides important information on a number of safety-related issues.

Risks varied across the different trial sites; clinical safety appreciation differs from site to site and probably from investigator to investigator. Also, the type of data and the way they are collected varies. While standard criteria exist [18], it would be useful to invest into harmonizing both the variables collected, the timing of assessment, and the grading of events, as well as the way data are reported and analysed.

One particular example is vomiting. Vomiting is a symptom of malaria, but can also be induced or made worse by treatment. In studies where treatment is given under direct observation it is possible to monitor the patients for the first hour post-dosing in case drug is vomited; this also allows re-administering the dose (in toto or in part) when so required, and distinguishing between early and late vomiting [19]. Only one study provided for this [11]; all other relied upon subjects or parent/guardians recall of episodes occurring in between visits.

Multivariate logistic regression analysis, as well as the comparison of TEAEs and AEs, pointed to the importance of looking to the considerable background noise generated by malaria itself (pretreatment and recurrence) as well as other diseases and conditions. For instance, the risk of GI events in general (and more specifically anorexia and diarrhoea) and other AEs (weakness) was higher in patients with recurrent malaria; cough (indicative of non-malaria infections) increased with follow-up time; older age was associated with a lower risk of most AEs.

It is important to distinguish the signs/symptoms of malaria from those that may be caused by the treatment. Assessing the drug-event relationship is highly subjective and prone to bias. Recording the occurrence and severity of (a defined set of) events before treatment is administered allows a better appreciation of the real contribution of a treatment to a patient’s status. When this is done (in this set of studies this involved ca. half of the patients enrolled), it is possible to report TEAE - ie the occurrence post-treatment of a sign/symptom that was not present before treatment, or its worsening with treatment. This applies also to RCTs, where randomization is expected to even out the risks of events at enrolment, as one is interested not only on the relative (between-treatment) but also the absolute risks for toxicity. These analyses showed that the risks, when considering AEs over TEAEs, ranged from no difference (rare events like jaundice, tinnitus) to double (vomiting), triple (abdominal pain), five-fold (nausea, headache, dizziness) differences. The relationship with prevalence of signs/symptom at enrolment was greater for AEs compared to TEAEs (meaning that the definition of AE is less independent of the sign/symptoms related to the disease itself).

Traditionally, the main concern in malaria has been efficacy, which has driven treatment recommendations (and a living databases of efficacy has been created [20]). Safety and tolerability, instead, have been neglected. Now with generally efficacious, intensely used ACT, assessing safety risks has become as important as ever. Compared to pharmaco-vigilance, clinical trials offer the opportunity of closer, more detailed monitoring of events, but are unsuited for signal generation (rare events). Yet, monitoring and reporting on safety outcomes in clinical trials is more cumbersome and less standardized than for efficacy outcomes (and generally felt by trialists to be less appealing). Paradoxically, this has created situations whereby claims of toxicity based on dubious evidence can cause a treatment to be disliked, recommended against, or banned altogether.

In conclusion, this paper provides both (i) a comparative evaluation of the safety risks following various ACT on a large, representative sample of malaria patients; and (ii) a proposal for improved methods to assess safety risks.

## Abbreviations

ACT: Artemisinin Combination Therapy; AE: adverse event; AL: Artemether-Lumefantrine; AQ: amodiaquine; AS: artesunate; CQ: chloroquine; DP: Dihydroartemisinin-piperaquine; LM: Lagrange multiplier test; Max: maximum; Min: minimum; RCT: Randomized Controlled Trial; SP: sulphadoxine-pyrimethamine; TEAE: treatment emergent adverse event; WHO: World Health Organization.

## Competing interests

The authors declare that they have no competing interests.

## Authors’ contributions

JZ and PO designed the analysis, interpreted the data and prepared the manuscript. All authors read and approved the final manuscript.

## Disclaimer

P. Olliaro is a staff member of the WHO; the authors alone are responsible for the views expressed in this publication and they do not necessarily represent the decisions, policy or views of the WHO.
